# Development and optimization of spray‐dried functional oil microcapsules: Oxidation stability and release kinetics

**DOI:** 10.1002/fsn3.1684

**Published:** 2020-07-20

**Authors:** Hao Yue, Bin Qiu, Min Jia, Jie Liu, Jing Wang, Fenghong Huang, Tongcheng Xu

**Affiliations:** ^1^ Institute of Agro‐Food Science and Technology Shandong Academy of Agricultural Sciences/Shandong Provincial Food for Special Medical Purpose Engineering Technology Research Center/Key Laboratory of Agro‐Products Processing Technology of Shandong Province/Key Laboratory of Novel Food Resources Processing Ministry of Agriculture Jinan China; ^2^ China‐Canada Joint Lab of Food Nutrition and Health (Beijing) Beijing Technology & Business University (BTBU) Beijing China

**Keywords:** functional oil microcapsules, high amylose corn starch, response surface methodology, spray drying

## Abstract

This study aimed to optimize the microencapsulation method for a functional oil using high amylose corn starch (HACS) and assessed its structure and antioxidant capacity. The results showed that the optimal microencapsulation condition is achieved by using 28.5% of functional oil, 15.75% of HACS, and 57.86% of proportion of monoglyceride in emulsifier with 94.86% microencapsulation efficiency. Scanning electron microscopy and particle size measurement showed that the functional oil microcapsules were uniform size, smooth surface, spherical shape, and without cracks in the wall of the capsules. In vitro oil release of microencapsulates results showed that microencapsulated functional oil containing HACS has a better sustained release effect. The microcapsules containing HACS exhibited a lower lipid oxidation rate during storage. In conclusion, microencapsulation of HACS as wall material improved the stability of functional oil and this formulation of microcapsules was satisfactorily applied in powdered food for diabetic patients.

## Graphical abstract

Function oil microcapsules could be produced using high amylose corn starch as a wall material. Addition of high amylose corn starch does not affect the efficiency of microencapsulation. The release of function oil can be controlled by adding high amylose corn starch.

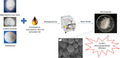

## 1 INTRODUCTION

Diabetes mellitus is an important threat to human life and health, and 90% of diabetic patients are type 2 diabetes mellitus (T2DM). Dr. Sanjay Basu estimated that the total number of T2DM will increase from 406 million in 2018 to 511 million in 2030 by analyzing data from the International Diabetes Federation (IDF) (Basu, McKee, Galea, & Stuckler, 2013; Wild, Roglic, Green, Sicree, & King, 2004). Diabetic patients have special physiological and nutritional needs, and the epidemiological studies had shown that high omega‐3 polyunsaturated fatty acid (ω‐3 PUFA) intake is inversely associated with diabetes mellitus (Adkins & Kelley, 2010; Endo & Arita, 2016; Martinez‐Fernandez, Laiglesia, Huerta, Martinez, & Moreno‐Aliaga, 2015; Tzang et al., 2009). In the past few decades, more and more studies have been done on functional oil. Functional oil is a kind of oil with special physiological functions such as improving metabolism of lipid and glucose, antithrombosis and improving cognitive ability, and mainly composed of oils rich in ω‐3 PUFA and ω‐6 PUFA (Day, Seymour, Pitts, Konczak, & Lundin, 2009). Previous studies have pointed out that the better ratio of ω‐6 PUFA to ω‐3 PUFA for diabetes control is 4–6:1 (Huang, Hou, Yeh, & Yeh, 2015; Kagohashi & Otani, 2010; Noori et al., 2011; Simopoulos, 2002, 2008). Nutrition guidelines for some countries had proposed that the proportion of MUFA in total fatty acids should be more than 30% (Kris‐Etherton et al., 2000; Sugano & Hirahara, 2000).

Although functional oil has many health benefits, there are safety problems associated with functional oil rich in ω‐3 PUFA. The main drawback of functional oils is the rapid oxidation of PUFAs, which can lead to the formation of toxic substances (Barroso, Pierucci, Freitas, Torres, & Rocha‐Leao, 2014; Binsi et al., 2017). Microcapsule technology is a mature industrial technology, and it has been widely used in food, medicine, and other fields through interdisciplinary research in recent years (Gharsallaoui, Roudaut, Chambin, Voilley, & Saurel, 2007). Among the various techniques used for microencapsulation, due to its simple operation, small footprint, and low cost, spray drying is the most widely used in the food industry (Tan, Chan, & Heng, 2005). The common wall materials are divided into three major categories: carbohydrates, proteins, and hydrophilic colloids (Ahn et al., 2008; Krishnan, Kshirsagar, & Singhal, 2005). Glucose index in diabetic patients fluctuates greatly when they intake carbohydrates, so the choice of wall materials is particularly critical in the embedding of functional oils. The Food and Drug Administration (FDA) indicated that high amylose corn starch (HACS) can reduce the risk of type 2 diabetes mellitus. Several studies reported that HACS intake significantly reduces the weight gain and lipid profiles of mice fed with high‐fat diet (Lee, Yoo, & Lee, 2012; Shimotoyodome, Suzuki, Fukuoka, Tokimitsu, & Hase, 2010). Previous researches confirmed that the application of microcapsule technology has positive effects on the relief of obesity or diabetes (Arifin et al., 2011; Li, Wu, & Dou, 2019; Mooranian, Negrulj, & Al‐Salami, 2016; Schneider et al., 2005). Hence, the functional oil microcapsules used HACS as wall materials may be highly advisable to control the condition of diabetic patients (Kieffer et al., 2016; Maki et al., 2012).

In addition to preventing oxidation, microencapsulation can also control the release of core materials ingredients (Koupantsis, Pavlidou, & Paraskevopoulou, 2016). In general, carbohydrates and proteins are not conducive to controlled release applications when used as wall materials (Cho, Shim, & Park, 2003). However, the content of resistant starch in HACS is relatively high, and the stability of microcapsules would be greatly increased when HACS was used as the wall material. Therefore, the purpose of the current study was to optimize the conditions for preparing functional oil microcapsules with HACS during spray drying. Besides, the structural, oxidative stabilization and sustained release effects of the microcapsules were also evaluated.

## 2 MATERIALS AND METHODS

### 2.1 Materials

Chia seed oil was provided by Aarhus Kars Oil Co., Ltd; peanut oil was provided by Shandong Jinsheng Grain and Oil Group Co., Ltd; and olive oil, maltodextrin, sodium caseinate, and HACS were purchased in local markets. All chemical reagents (analytical grade) were purchased from Sinopharm Chemical Reagent Co., Ltd.

### 2.2 Preparation of functional oil

Chia seed oil, peanut oil, and olive oil were used as raw materials, and the functional oil was prepared according to the ratio of Chia seed oil:peanut oil:olive oil = 1:8:1. The ratio of saturated fatty acids (SFA), monounsaturated fatty acids (MUFA) and PUFA in functional oils was 1:2:2, and the relative content ratio of ω‐6 PUFA to ω‐3 PUFA was 5:1. The samples were stored at 4°C until used.

### 2.3 Preparation of emulsions

The wall material which includes the maltodextrin, sodium caseinate, and HACS was dissolved in water (65°C). The functional oil was emulsified at 65°C. Functional oil and wall materials were emulsified for 5 min at 8,950 *g*.

### 2.4 Spray drying of emulsions

After a stable emulsion was obtained by three times of 30 MPa homogenization (Changzhou Homogenizer Machinery Corporation, Ltd., GJB 8‐20), spray drying was employed using a small‐scale spray dryer (Buchi, model 290). The operating conditions were as follows: Inlet temperature was 185°C, outlet temperature was about 90°C, and feed rate was 5 ml/min.

### 2.5 Microencapsulation efficiency (MEE)

The MEE was determined by the following formula by Trindade (Trindade & Grosso, 2000).

$$\text{MEE}\%=\frac{\text{total}\;\text{oil}-\text{extractable}\;\text{oil}}{\text{total}\;\text{oil}}\times 100$$

### 2.6 Microcapsule formulation optimization

Design‐Expert 8.0.6.1 software was used to design the experiments. Response surface methodology (RSM) by a 3‐factor‐3‐level Box–Behnken design was used to investigate the variation of MEE with respect to formula parameters including HACS, functional oil content, and percentage of monoglyceride in emulsifier. The variables and their concentration ranges are as follows: core material (functional oil) content (*X*_1_) from 20% to 40% (w/w), proportion of HACS in wall polymers (*X*_2_) from 10% to 30% (w/w), and proportion of monoglyceride in emulsifier (*X*_3_) from 50% to 70%. The actual variable was coded to facilitate multiple regression analysis (Table 1).

**TABLE 1:** Coded levels for independent variables used in experimental design for microencapsulation of FO with high amylose corn starch

Variables	Coded *X_i_*	Coded level		
		−1	0	1
Concentration of function oil (g)	*X*_1_	20	30	40
Concentration of high amylose corn starch (g)	*X*_2_	10	20	30
Proportion of monoglyceride in emulsifier (%)	*X*_3_	50	60	70

### 2.7 Characterization of the microcapsules

#### 2.7.1 Moisture

AOAC method was used for determined moisture content of microcapsules (AOAC, 2000).

#### 2.7.2 Determination of bulk density

Accurately weighed 5 g of functional oil microcapsule samples, loaded them into graduated cylinders, measured and calculated their volume, and calculated the mass per unit volume of microcapsules.

#### 2.7.3 X‐ray diffraction

The microcapsule samples (0.5 g) were weighed and placed in the sample box of fully automatic X‐ray diffractometer. Measuring conditions were as follows: radiation source: Cu, Kα; voltage: 40.0 kV; current: 30.0 mA; scanning rate: 2°/min; scanning range: 5°–90°; and scanning mode: continuous.

#### 2.7.4 Fourier transform‐infrared spectroscopic analysis (FT‐IR)

The FT‐IR was used to analyze functional oil, HACS, maltodextrin, sodium caseinate, and functional oil microcapsules.

##### Solid sample analysis

Accurately weigh 1 mg of microcapsule samples. Potassium bromide mass ratio = 1:100. After mixing and grinding in a mortar, the tablets were compressed and scanned in the range of 400–4,000 cm^−1^.

##### Liquid sample analysis

A small amount of functional oil samples was pipetted by a dropper and uniformly applied to the prepared potassium bromide tablet for infrared analysis, and scanned in the range of 400–4,000 cm^−1^.

#### 2.7.5 Particle size measurement

The functional oil microcapsule samples were uniformly dispersed in distilled water at room temperature. The particle size distribution of the functional oil microcapsules was determined by BT‐2001 laser particle size analyzer, and data sampling analysis was performed.

#### 2.7.6 Morphological study

The surface and internal morphology of functional oil microcapsules was observed using a scanning electron microscope (SEM). Attach one side of the double‐sided tape to the sample stage, then stick small amount of microcapsule samples on it, and spray the gold (thickness 100 μm). The structure is observed and photographed at an acceleration voltage of 10 kV.

### 2.8 Storage stability of microcapsules

The functional oil and microcapsules were placed in 62°C ± 2°C oven for accelerated oxidation experiments. The change in peroxide value was measured every 24 hr during the heating period of 7 days.

### 2.9 Oil release kinetics during in vitro digestion

Simulated gastric fluid (SGF) and simulated intestinal fluid (SIF) were prepared according to the method provided by the United States Pharmacopoeia (US Pharmacopeia, 2000). The appropriate amount of functional oil microcapsules was initially dissolved in the SGF for 100 min at 37°C and 100 rpm/min. Further, SIF was added and intestinal digestion was continued for 150 min under similar conditions.

## 3 RESULTS

### 3.1 Optimization of microcapsule sample by RSM

**FIGURE 1:**
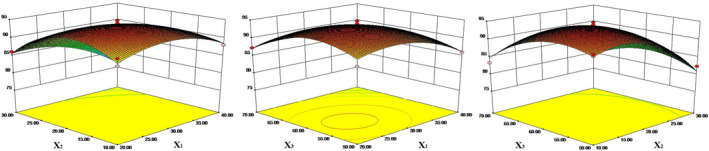
Effect of the interaction of factors on the MEE

The RSM was used to evaluate the impact of multiple parameters on response variables. Based on the coding levels of the three independent variables (Table 1), a three‐factor BBD was used to obtain 17 simplified experimental set (Table 2). The functional oil concentration, the proportion of HACS in the wall material, and the proportion of monoglyceride in emulsifier were investigated in the ranges of 20%–40% (w/w), 10%–30% (w/w), and 50%–70% (w/w), respectively. From the regression coefficients and *p*‐value, the order of influence of various factors on the embedding efficiency of functional oil microcapsules was as follows: proportion of HACS (*X*_2_) > functional oil concentration (*X*_1_) > proportion of monoglyceride in emulsifier (*X*_3_), and the impact of various factors on the embedding efficiency is extremely significant (*p* < .01) (Table 3), and the MEE response surface graphs are shown in Figure 1. The Design‐Expert 8.0.6 was used to statistically analyze the results determined by the seventeen conditions, and a quadratic regression equation was established as follows: 

**TABLE 2:** Central composite design for the optimization of microencapsulation

Run	A	B	C	MEE%
1	0	0	0	93.76
2	−1	1	0	86.1133
3	−1	0	−1	88.75
4	0	1	1	78.78
5	1	−1	0	88.02
6	0	1	−1	82.34
7	1	0	−1	86.03
8	0	−1	1	83.33
9	−1	0	1	87.27
10	1	0	0	94.69
11	−1	−1	0	90.69
12	0	0	0	93.93
13	1	1	0	75.19
14	1	0	1	83.06
15	0	0	0	94.68
16	0	−1	−1	92
17	0	0	0	91.97

**TABLE 3:** Values of regression coefficients calculated for the functional oil microencapsulation

Source	*R* ^2^	*df*	Mean square	*F* value	*p*‐value	Significance
Model	511.57	9	56.84	20.59	.0003	**
*X*_1_	52.65	1	52.65	19.07	.0033	**
*X*_2_	124.95	1	124.95	45.26	.0003	**
*X*_3_	34.78	1	34.78	12.6	.0094	**
*X*_1_ *X*_2_	17.03	1	17.03	6.17	.042	*
*X*_1_ *X*_3_	0.56	1	0.56	0.2	.6674	
*X*_2_ *X*_3_	6.53	1	6.53	2.36	.168	
$X_{1}^{2}$	46.38	1	46.38	16.8	.0046	**
$X_{2}^{2}$	126.62	1	126.62	45.86	.0003	**
$X_{3}^{2}$	74.62	1	74.62	27.02	.0013	**
Residual	19.33	7	2.76			
Lack of fit	14.39	3	4.8	3.89	.1112	
Pure error	4.93	4	1.23			
Cor total	530.89	16				
*R* ^2^ = .9636; $R_{\text{Adj}}^{2}$ = .9168						

* Significant difference (*p* < .05).

** Very significant difference (*p* < .01).

The optimal formula for the preparation of functional oil microcapsules was 28.5% of functional oil, 15.75% of HACS, and 57.86% of proportion of monoglyceride in emulsifier. The MEE of functional oil microcapsules was theoretically 95.01% under these conditions. Repeated experiments showed that the average MEE of functional oil microcapsules was actually 94.86%, which was close to the theoretical optimal value and good repeatability. Furthermore, the models explain 96.3% of the variability in the responses.

### 3.2 Scanning electron microscopy (SEM)

**FIGURE 2:**
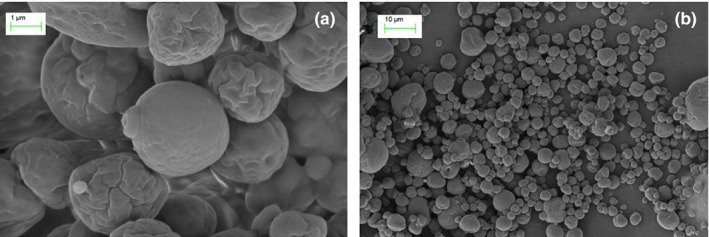
Microscopic images of functional oil emulsions: Micrographs at (a) 1,000 magnification and (b) 25,000 magnification

As shown in Figure 2, SEM was used to observe the morphology of microcapsules under optimized conditions. MEFO at the optimized condition (94.86% in MEE) showed approximately spherical, exhibited smooth and free of pores, the cyst wall is relatively complete, compact and without apparent fissures or cracks. Moreover, the shape of MEFO appeared to be slightly rough and concave on the surface due to instability during the drying process. Teixeira et al. reported that depressions and roughness of the surface are more prevalent in small particles than in larger ones, which indicated that the microcapsules expanded into a round shape and then wall material solidified (Teixeira, Andrade, Farina, & Rocha‐Leao, 2004). The higher temperature easily caused the rapid solidification and depression of the wall material, and it was difficult to form small particles with smooth surface. This is commonly characterized of high‐temperature spray‐dried microcapsule product, which is also observed by Tonon et al. in the microencapsulation of flaxseed oil (Tonon, Grosso, & Hubinger, 2011).

### 3.3 Moisture content and bulk density

Moisture content of MEFO is a parameter closely related to oil oxidation. The average moisture content of MEFO was 1.88% (Table 4). The reason for the low moisture content in MEFO was mainly the higher hydrophobicity imparted by HACS in the wall material. Liu et al. reported that the moisture level in flaxseed oil microencapsulated with gum arabic by spray drying was 3.17% (Liu, Low, & Nickerson, 2010). Tonon et al. found that lower moisture levels (0.57%–1.91%) in flaxseed oil microencapsulated with modified starch by spray drying (Tonon et al., 2011). Bulk density is a very important indicator in the industrial production of microcapsule products. Suitable bulk density is beneficial to reduce the processing costs of the product. The bulk density of MEFO was 0.36 g/ml, which is slightly higher than other similar oil microcapsules, indicating that it has good properties such as flowability (Asensio et al., 2017; Li & Shi, 2018; Li et al., 2013).

**TABLE 4:** Physicochemical and oil release properties of functional oil microcapsules

Parameter	Value	Parameter	Value
MEE (%)	94.86 ± 0.72	Surface oil (g/100 g)	1.32 ± 0.05
Moisture content (%)	1.88 ± 0.03	Total oil (g/100 g)	25.73 ± 0.07
Bulk density (g/cm^3^)	0.36 ± 0.05	Solubility (g/100 g)	92.83 ± 0.52
Oil released by gastric fluid (%)	14.9 ± 0.19	Oil released by intestinal fluid (%)	65.2 ± 0.61

### 3.4 FT‐IR spectra of microcapsules

**FIGURE 3:**
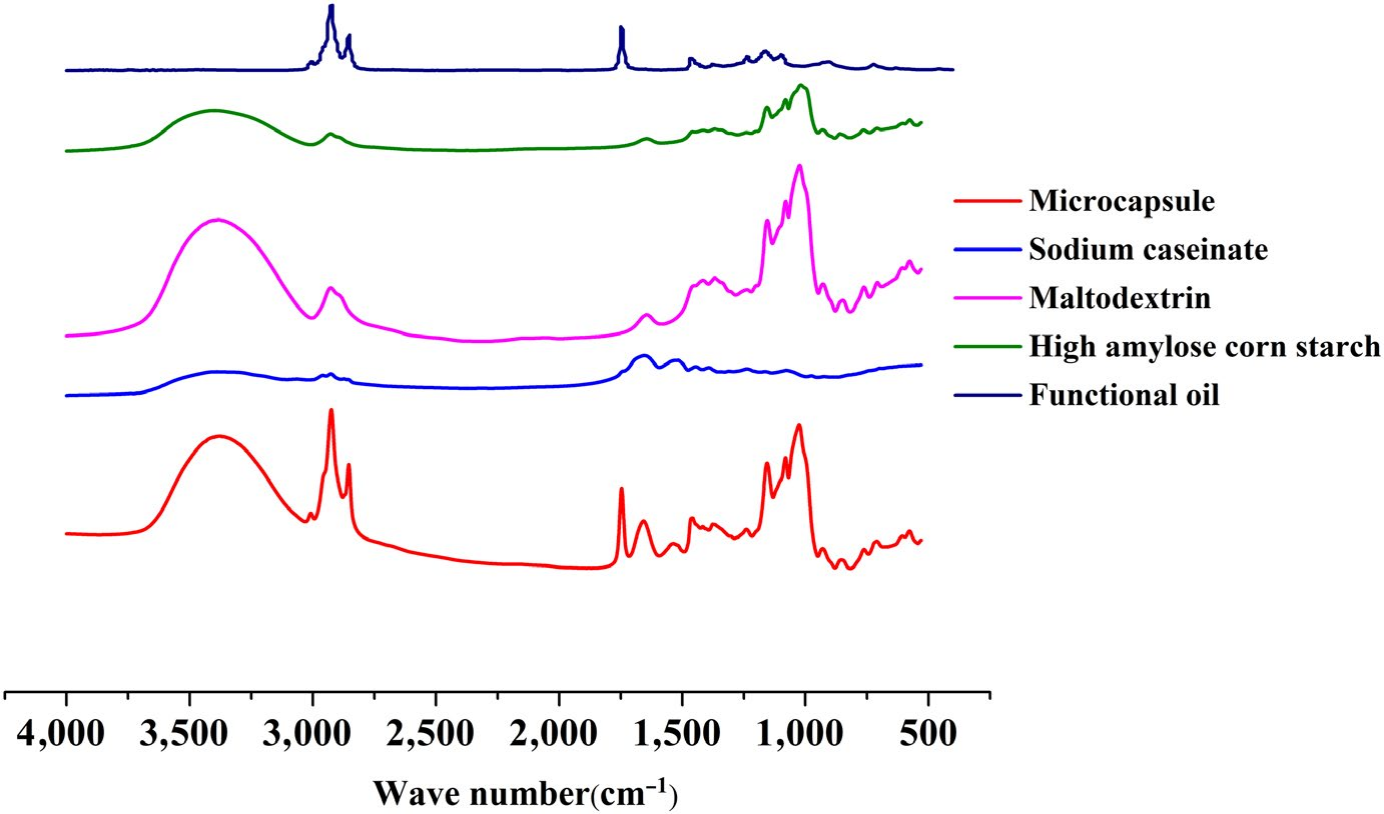
FT‐IR profile of functional oil microcapsules

FT‐IR spectroscopy analysis was used to explore the interaction of wall materials and functional oil in MEFO (Figure 3). At the 3,600–3,300 cm^−1^, since maltodextrin and HACS a large amount of OH. When applied to MEFO, this characteristic peak also exists obviously. In the region from 1,800 to 1,400 cm^−1^, the characteristic peak may be caused by the stretching vibration peak caused by C=C. The characteristic peaks of functional oil appeared at 2,990 cm^−1^, which was caused by the stretching vibration of the = C‐H bond in functional oil. Similarly, similar characteristic peaks existed in the infrared spectra of functional oil microcapsules at the same location. From the spectral changes observed in current study, the main characteristic peaks of wall materials and functional oil were shown on the spectrogram of microcapsule products, but were weakened to varying degrees, which may be due to the interaction between the wall polymers and functional oil.

### 3.5 Powder X‐ray diffraction

**FIGURE 4:**
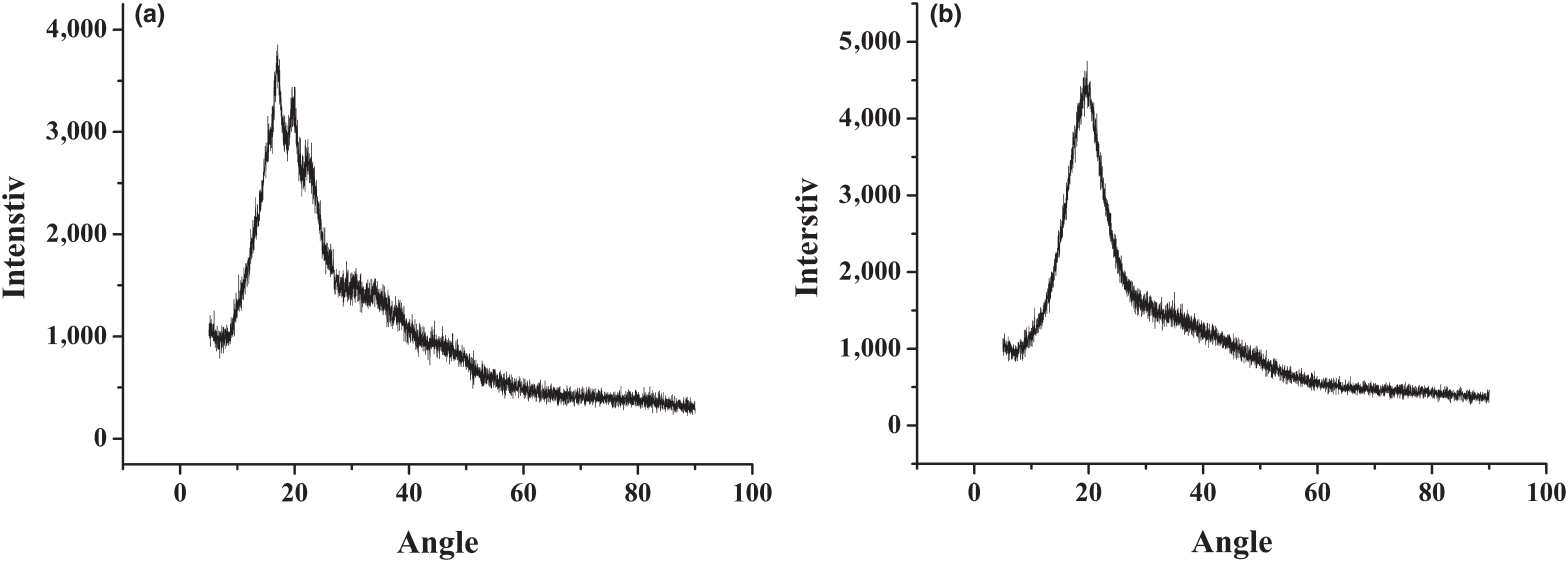
X‐ray diffraction analysis, used to investigate sample crystallinity: (a) The semicrystalline structure of HACS and (b) the amorphous structure of microcapsules of functional oil

As shown in Figure 4, the microcapsule samples were amorphous; however, the HACS has a semicrystalline structure. The cause of this phenomenon may be that the oil interacted with the HACS hydrophobic group, resulting in the disappearance of the crystal structure. The highly ordered state of the molecules caused sharp and clear peaks in the crystalline material, while the disordered display of amorphous molecules was responsible for the appearance of diffuse and large peaks in amorphous materials (Yu, 2001). The amorphous structure in the microcapsule promotes high dispersibility in water, which is one of the important properties of its application in the food industry.

### 3.6 Particle size measurement

The particle size measurement showed that the minimum range of microcapsules appeared was 0.29–0.36 μm, and the particle size distributions of D10, D50, and D90 were 0.795, 3.871, and 14.45 μm, respectively. The average particle size was 6.064 ± 0.38 μm, and the smaller or larger diameter particles were less. The average particle size in the current study is lower than that observed in other oil microcapsules studies. Pu, Bankston, and Sathivel (2011) used sodium caseinate as the wall material and flaxseed oil as the core material to obtain microcapsules with an average diameter of 25.1 μm. Reineccius (1989) found that particles within 100 mm increased the solubility of the powder, so smaller particle sizes were important for microcapsules.

### 3.7 Effect of SGF and SIF on oil release

**FIGURE 5:**
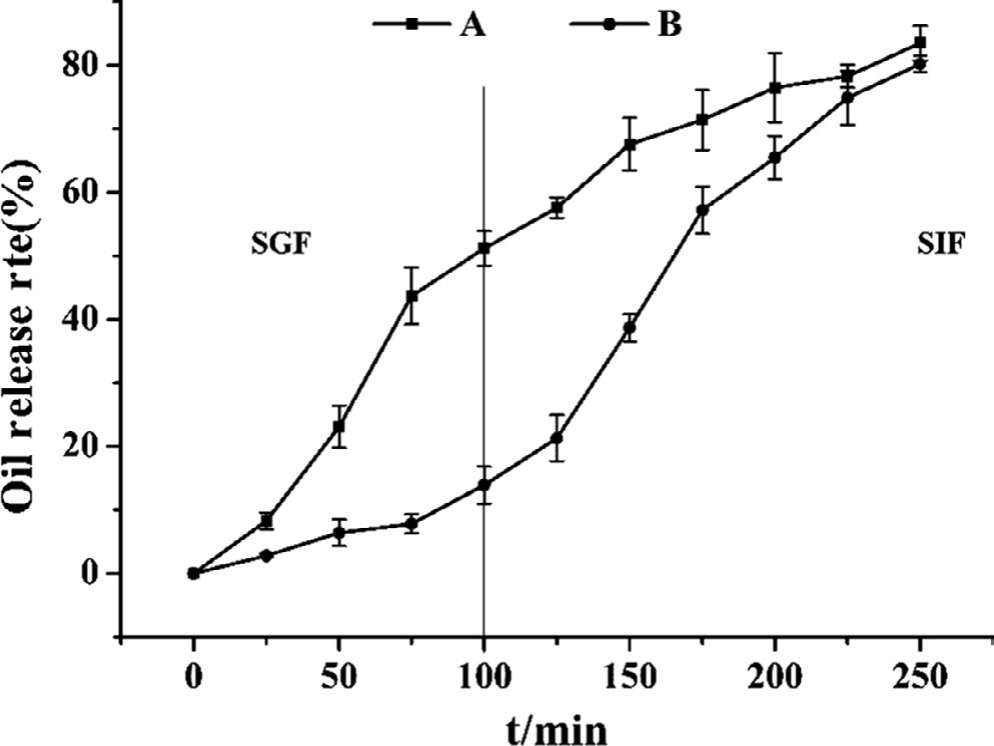
In vitro release profile of functional oil microcapsules: (a) MEFO without HACS and (b) MEFO

In order to evaluate the release pattern of microcapsules in vitro, simulated digestion was used to test the stability and release behavior of microcapsules during gastrointestinal transit. In vitro simulated digestion is mainly divided into two parts: simulated digestion in the stomach and simulated digestion in the intestine. Generally, food was withheld for 100 min after entering the stomach and then entered the small intestine for further digestion for 150 min. As shown in the Figure 5, MEFO exhibited better sustained release effect than MEFO without HACS. In the first 100 min of gastric digestion, the microcapsules were in the acidic environment of gastric acid as a whole. The wall materials of microcapsules had a controlling effect on the release of gastric acid. Microencapsulated core materials containing HACS in wall materials could only release <20% within 100 min of digestion, but the release rate of microencapsulated core material without HACS reached 50% within 100 min of digestion. Although strong acidic environment had a certain threat to the protein in microcapsules, the high viscosity of HACS and emulsifier made the wall materials of microcapsules more intact and prevented the release of core materials. When digested in small intestine for 100–250 min, the core material of functional oil microcapsules was released in large quantities due to the decomposition of trypsin and the acid‐alkaline environment. At the end of 250 min, the total release of microcapsules with different formulations was similar, and more than 80% of functional oil was released.

### 3.8 Storage stability analysis

**FIGURE 6:**
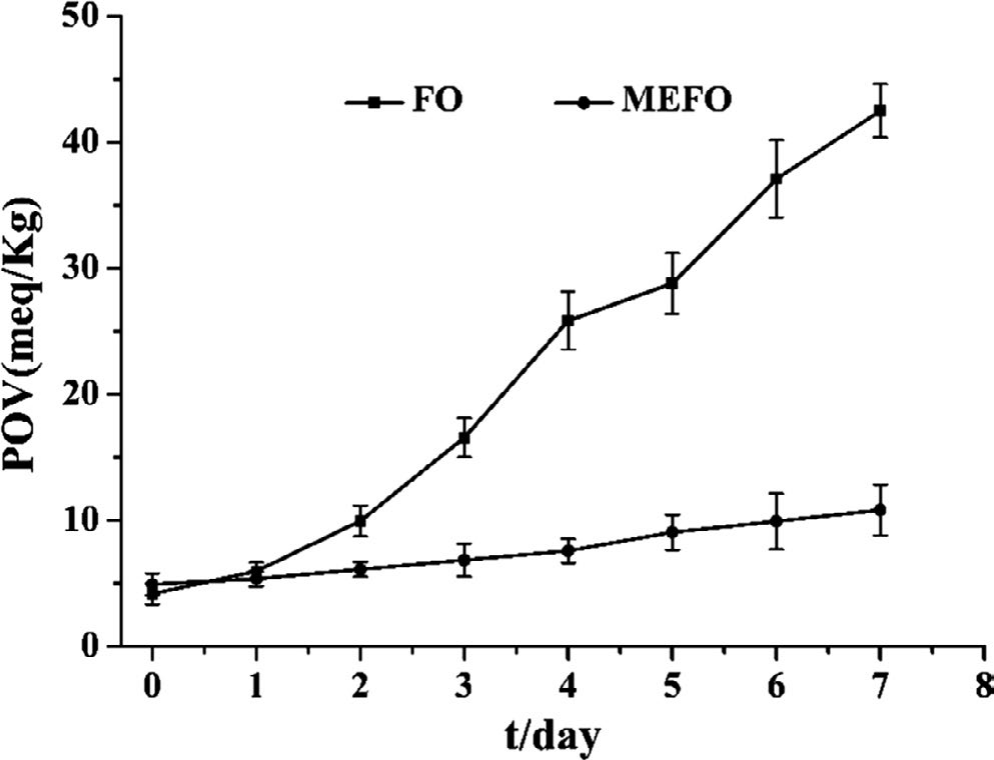
Changes in POVs of functional oil microcapsules and during accelerated storage study

In order to ascertain the antioxidant stability of microcapsules, the change of peroxide value (POV) in the outer free oil of and inner oil of MEFO during the accelerated storage of the oven at 62 ± 2°C was observed. As shown in Figure 6, initial POV of functional oil was 4.0 meq/kg and initial POV of functional oil after microencapsulation was 4.9 meq/kg. This result indicated that the high temperatures during spray drying caused the oil to oxidize to some extent before getting microencapsulated. However, the oil oxidation was greatly retarded by microencapsulation during stored. The functional oil was similar to the oxidative trend of MEFO at first, but the POV content increased significantly after 2 days of stored. At the end of the accelerated oxidation experiment, the peroxide values of functional oil and microcapsules increased to 42.5 and 10.3 meq/kg, respectively. These results indicated that the MEFOS has superior oxidative stability, which prevented diffusion of functional oil and against oxygen from contact with the core material. Previous studies found the similar results in microencapsulation. Asensio et al. (2017) reported that the composition of wall material is critical in antioxidation. Kolanowski, Jaworska, Weiβbrodt, and Kunz (2007) found that microencapsulated fish oil oxidized rapidly in the presence of air, which was improved after microencapsulation.

## 4 CONCLUSION

The purpose of the current study was to demonstrate the feasibility of HACS as wall material for the production of functional oil microcapsules by spray drying and optimize the microencapsulation condition. The optimal microencapsulation condition obtained through RSM was 28.5% of functional oil, 15.75% of HACS, and 57.86% of proportion of monoglyceride in emulsifier. The MEE of functional oil microcapsules was 94.86% under these conditions. The results of SEM and particle size measurement showed that the addition of HACS did not affect the uniformity and surface morphology of the particles. FT‐IR spectroscopy analysis of the microcapsules confirmed the interaction of HACS with wall material and functional oil. The results of POV during accelerated storage showed the protective effect of microencapsulation on functional oil. In vitro simulated digestion experiment, the release of functional oil can be controlled by adding HACS. In conclusion, current finding shows that incorporation of HACS in functional oil microcapsules in spray drying may be advocated, the process of encapsulation itself not only improved the oxidative stability of the oils, but also enhance the nutritional value of microcapsules. The use of this microencapsulation design may result in increased utilization of functional oil in food industry, which may help improve the health of diabetic patients.

## CONFLICT OF INTEREST

All authors declared that they have no personal or financial conflicts of interest.
